# Efficacy of Various Scoring Systems for Predicting the 28-Day Survival Rate among Patients with Acute Exacerbation of Chronic Obstructive Pulmonary Disease Requiring Emergency Intensive Care

**DOI:** 10.1155/2017/3063510

**Published:** 2017-05-25

**Authors:** Zhihong Feng, Tao Wang, Ping Liu, Sipeng Chen, Han Xiao, Ning Xia, Zhiming Luo, Bing Wei, Xiuhong Nie

**Affiliations:** ^1^Department of Respiratory Medicine, Xuanwu Hospital, Capital Medical University, Beijing, China; ^2^Department of Emergency Medicine, Beijing Chaoyang Hospital Affiliated to Capital Medical University, Beijing, China; ^3^Department of Physiology and Pathophysiology, Capital Medical University, Beijing, China; ^4^Department of Public Health, Capital Medical University, Beijing, China

## Abstract

We aimed to investigate the efficacy of four severity-of-disease scoring systems in predicting the 28-day survival rate among patients with acute exacerbation of chronic obstructive pulmonary disease (AECOPD) requiring emergency care. Clinical data of patients with AECOPD who required emergency care were recorded over 2 years. APACHE II, SAPS II, SOFA, and MEDS scores were calculated from severity-of-disease indicators recorded at admission and compared between patients who died within 28 days of admission (death group; 46 patients) and those who did not (survival group; 336 patients). Compared to the survival group, the death group had a significantly higher GCS score, frequency of comorbidities including hypertension and heart failure, and age (*P* < 0.05 for all). With all four systems, scores of age, gender, renal inadequacy, hypertension, coronary heart disease, heart failure, arrhythmia, anemia, fracture leading to bedridden status, tumor, and the GCS were significantly higher in the death group than the survival group. The prediction efficacy of the APACHE II and SAPS II scores was 88.4%. The survival rates did not differ significantly between APACHE II and SAPS II (*P* = 1.519). Our results may guide triage for early identification of critically ill patients with AECOPD in the emergency department.

## 1. Introduction

Early identification of patients at high risk for severe disease or death enables clinicians to promptly initiate aggressive treatment and thereby save lives. Many severity-of-disease scoring systems have been developed to allow doctors to quickly and simply identify patients requiring urgent care. These systems use clinical data typically recorded at admission, like heart rate, blood data, and temperature, and the scores yielded by these systems have prognostic value.

Acute Physiology and Chronic Health Evaluation (APACHE) II is the most commonly used severity-of-disease assessment system. It comprehensively reflects the disease severity in critically ill patients and can predict patient survival, prognosis, and hospitalization and medical costs, to some extent. Although APACHE II is designed for intensive care unit (ICU) patients and is mostly used for prognostic evaluation of such patients, it is increasingly being used for emergency department patients and has been proven to yield excellent results for clinical prediction of sepsis in these patients [1–4]. Recently, it has also gained popularity for prognostic evaluation of systemic diseases [5]. Dynamic trends of the APACHE II score reflect disease evolution, enable timely clinical interventions, and guide amendments in healthcare plans. However, this system is very complex and has not been proven to correctly reflect the severity of disease in different affected organs in the case of multiorgan disease. Additionally, it has low specificity, does not yield exact quantitative assessments, and is not uniform or standardizable [6]. On the other hand, the Simplified Acute Physiology Score (SAPS) II system is simpler as it combines the basic components of the APACHE scoring system with logistic regression analysis. Recent reports show that the SAPS II system can be used to evaluate the condition of critically ill patients and predict mortality.

In 1996, Vincent et al. proposed the Sepsis-Related Organ Failure Assessment (SOFA) system, which aims to continuously reflect the dynamic development of multiorgan dysfunction syndrome, from mild to severe [7]. The SOFA score may reflect the degree of organ dysfunction in patients with severe disease and reportedly shows good correlation with prognosis [8]. Although it was developed in relation to sepsis, the system's scope of application extends far beyond septic shock. It has demonstrated value for assessing multiorgan dysfunction syndrome induced by noninfectious causes, for example, stem cell transplantation or burns [9]. Similarly, the mortality in emergency department sepsis (MEDS) score proposed by Shapiro et al. [10] is mostly used to assess the risk of mortality in patients with sepsis in the emergency department. The MEDS system is advantageous because it includes few parameters and yields a fixed value; additionally, its assessment of 28-day survival rate in patients at risk of infection in the emergency room is very accurate [10, 11]. It predicts this rate in patients with suspected infection, sepsis, and severe sepsis and is useful for 1-year mortality assessment in patients with suspected infection in the emergency room [11–13].

With its high prevalence, morbidity, and mortality, chronic obstructive pulmonary disease (COPD) imposes an increasing economic and social burden worldwide. Acute exacerbation is an important factor related to the mortality associated with COPD, and it causes intractable problems for clinical treatment. A study showed that over the course of a year, about 57% COPD patients required emergency treatment and 13% COPD patients required over 6 visits to the emergency department [14]. It is known that critically ill patients in the emergency department have a high mortality rate [15], and a study reported that acute exacerbation of COPD (AECOPD) accounted for 22.14% (441 cases) of 1992 such patients.

To our knowledge, no previous study has been conducted to identify the ideal system to triage patients with AECOPD requiring emergency care. Thus, the present study aimed to examine the efficacy of the four abovementioned severity-of-disease scoring systems in predicting the 28-day survival rate in patients with AECOPD. Our findings may guide special rescue strategies in order to prevent early mortality among such patients in the emergency department.

## 2. Materials and Methods

### 2.1. Study Site and Patient Recruitment

**Figure 1:**
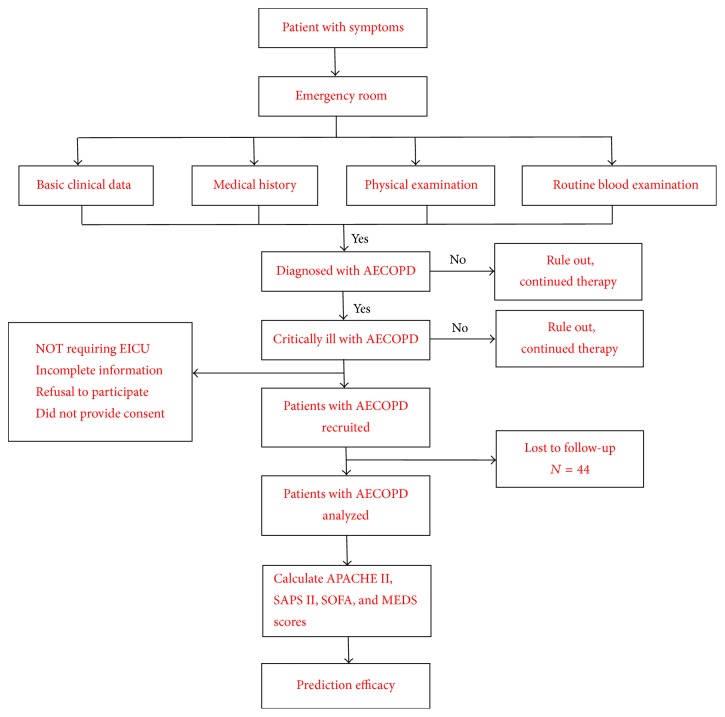
Flow chart of inclusion and recruitment workup (APACHE II: Acute Physiology and Chronic Health Evaluation; SAPS II: the Simplified Acute Physiology Score II; SOFA: Sepsis-Related Organ Failure Assessment; MEDS: mortality in emergency department sepsis).

We prospectively analyzed patients with AECOPD who were admitted to the intensive care unit of the emergency department of Xuanwu Hospital, the Capital Medical University, between October 2012 and 2014. We included (1) all patients who met the diagnostic criteria for COPD [16] and (2) those with AECOPD (3) who were in a critical condition and needed emergency diagnosis and intensive care treatment [16]. The exclusion criteria were (1) bronchial asthma, community-acquired pneumonia, bronchiectasis or interstitial lung disease, and other respiratory system diseases; (2) grave illness not requiring emergency intensive care treatment; (3) incomplete information; (4) and refusal to participate or nonprovision of signed informed consent. This study was approved by the ethics committee of Xuanwu Hospital, and all clinical investigations have been conducted according to the principles expressed in the Declaration of Helsinki. All participants provided signed informed consent. The patient records/information was anonymized and deidentified prior to analysis (Figure 1).

### 2.2. Collection of Blood Samples and Patient Data

The starting point of patient follow-up was the point of admission for emergency intensive care, and the end point was 28 days after admission. The 28-day survival rate represents the number of patients who were alive at the end point. Similarly, the starting point of observation was the AECOPD event, and the end point was death within 28 days (all-cause mortality). For follow-up of patients who left the hospital, a specialist contacted them on the telephone at the end point. Patient data, including name, gender, age, height, weight, body mass index, Glasgow coma scale (GCS) score, and medical history, were recorded in detail. Before any intervention was performed, vital signs were recorded, and routine blood examination, including clotting function, blood gas analysis, and biochemical parameters, was conducted; additionally, C-reactive protein levels were determined. The four severity-of-disease indicators were calculated at 24 h after admission to the hospital, that is, APACHE II, SAPS II, SOFA, and MEDS scores.

### 2.3. Statistical Analysis

Demographic and clinical characteristics are presented as mean ± SD for continuous variables and as frequencies for categorical variables. Continuous variables were compared using* t*-tests or the Mann-Whitney* U* test, and categorical variables were compared using *χ*^2^ tests or the Fisher exact test. Patients were divided into the death and survival groups depending on their outcomes at 28 days after admission to the intensive care unit of the emergency department. Student's* t*-tests and analysis of covariance were used to compare the four severity-of-disease indicators among groups. Multivariate logistic regression was used to compare parameters between the death and survival groups, including variables that differed significantly between the groups at the baseline and the scores of the four severity-of-disease indicators. Confounders were included in the multivariate logistic regression using the backward stepwise method if the *P* value was equal to or less than 0.05.

To avoid overlap in the same regression model, which could result in collinearity between the variables, logistic regression models including each scoring method and baseline variables were established, and variables were selected using the backward stepwise regression method.

Receiver operating characteristic (ROC) curves were drawn using the outcomes to indicate significant differences in the four severity-of-disease indicators between groups. Further, the area under the ROC curve (AUC) was compared among the scoring methods. All significance tests were two-sided, and *P* values <0.05 were considered statistically significant. Analyses were conducted using SPSS statistical software version 20.0 (SPSS Inc., Chicago, Illinois, USA).

## 3. Results

### 3.1. Clinical Data of Patients with AECOPD in the Survival and Death Groups

A total of 426 patients with AECOPD met the criteria for receiving emergency intensive care. However, 44 patients were lost to follow-up (follow-up rate = 89.7%), and 382 patients were finally included in the study. Forty-six patients (26 men and 20 women; average age, 77.43 ± 10.96 years; age range, 46–90 years) were included in the death group: 39 died during hospitalization, while 7 died after leaving the hospital (all within 28 days of emergency hospitalization for AECOPD), and the mortality rate was 12.04%. The survival group comprised 336 patients (214 men and 122 women; average age, 62.80 ± 14.16 years; range, 36–94 years).  Table 1 shows a comparison of clinical data between the survival and death groups. Compared to patients in the survival group, those in the death group had a significantly higher GCS score and frequency of comorbidities including hypertension, coronary heart disease, heart failure, arrhythmia, anemia, fracture leading to bedridden status, and tumor and were older (*P* < 0.05 for all).

### 3.2. Comparison of Severity-of-Disease Indicators between the Survival and Death Groups of Patients with AECOPD

Univariate analysis show that, compared to the survival group, the death group showed significantly higher APACHE II (*P* < 0.001), SAPS II (*P* < 0.001), and MEDS scores (*P* = 0.011). However, no significant differences were found in the SOFA score between the groups (*P* = 0.055). Analysis of covariance including age, gender, renal inadequacy, hypertension, coronary heart disease, heart failure, arrhythmia, anemia, fracture leading to bedridden status, tumor, and GCS scores showed significantly higher scores in the death group compared to the survival group for the four severity-of-disease indicators. Additionally, they were all significantly different between the groups (Table 2).

### 3.3. Logistic Regression Analysis of Severity-of-Disease Scoring Systems for Predicting 28-Day Survival Rate

Table 3 shows the results of logistic regression analysis of the four severity-of-disease scoring systems. Models I and II showed that the APACHE II and SAPS II scores, with AUCs of 89.9% and 86.2%, respectively, significantly influence 28-day survival (*P* < 0.001 for both). In other words, both APACHE II and SAPS II scores had individual predictive value for 28-day survival in patients with AECOPD. Model IV also showed that the APACHE II (*P* < 0.001) and SAPS II scores (*P* < 0.001) had similar predictive power for 28-day survival, with a mean AUC of 88.4%.

### 3.4. Use of ROC Curves for Predicting 28-Day Survival Rate of Patients with AECOPD

**Figure 2:**
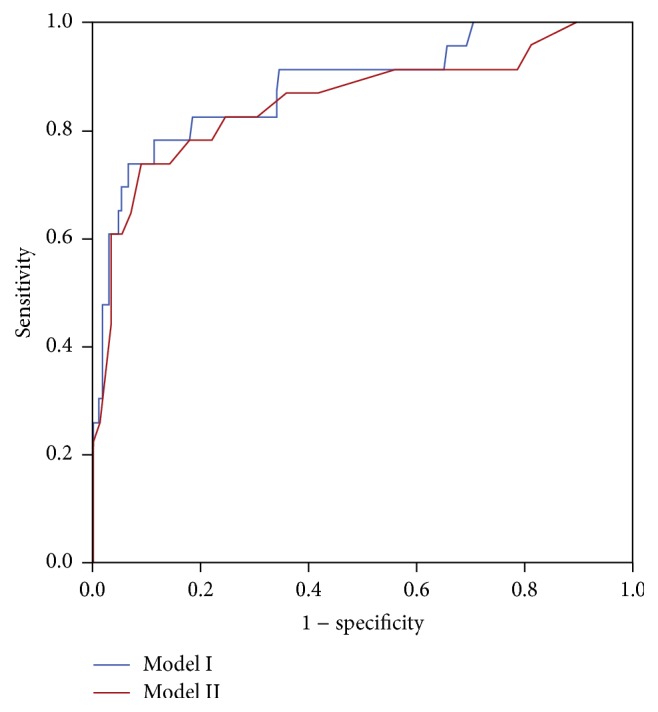
ROC curves of APACHE II and SAPS II scores predicting the 28-day survival rate.

The AUCs of Model I and Model II were 0.899 ± 0.030 (95% CI, 0.856–0.942) and 0.862 ± 0.043 (95% CI, 0.811–0.912), respectively, and these values showed a significant difference (*P* < 0.001). The best threshold value of the APACHE II score for predicting prognosis was 17 points, and its sensitivity and specificity were 69.6% and 91.7%, respectively. For patients with AECOPD requiring emergency critical care, those with APACHE II scores ≥ 17 showed a 28-day survival rate of 56.76%. In terms of the SAPS II score, the best threshold value for predicting prognosis was 32 points, and its sensitivity and specificity were 83.9% and 91.1%, respectively. For patients with SAPS II scores ≥ 32 points, the 28-day survival rate was 52.78%. Thus, although the AUC of Model II was lower than that of Model I, the ability of these indexes to predict the 28-day survival rate was similar and showed no significant difference (*P* = 1.519) (Figure 2, Table 4).

## 4. Discussion

Emergency departments care for a large number of patients on a daily basis. Clinical manifestations greatly influence the assessment of critically ill patients, and patients are mostly assessed on the basis of doctors' subjective knowledge and experience. Doctors often pay more attention to apparently severe conditions and may easily overlook patients whose clinical manifestations are mild at first but rapidly deteriorate to alarming severity. Therefore, a systematic and scientific approach for patient examination is essential in order to ensure timely and appropriate treatment for critically ill patients with atypical clinical manifestations. AECOPD poses a major challenge for emergency doctors in China given the high incidence and mortality rate of this condition and the fact that most critically ill patients with AECOPD in China seek treatment in the emergency department. Despite this, most studies conducted to date regarding the prognosis of patients with AECOPD have been conducted in the respiratory department or respiratory ICU [17, 18]. To our knowledge, the present study is the first to analyze the early prognosis of patients with AECOPD in the emergency department.

Our results showed that, compared to the patients in the survival group, those in the death group had a significantly higher GCS score and frequency of comorbidities including hypertension, coronary heart disease, heart failure, arrhythmia, anemia, fracture leading to bedridden status, and tumor and were older (*P* < 0.05 for all). Thus, these factors seem to be important predictors of the 28-day survival rate among patients with AECOPD who seek emergency intensive care. Further, compared to the survival group, the death group had significantly higher APACHE II, SAPS II, SOFA, and MEDS scores. These findings confirmed that four scores are useful for prognosis prediction in critically ill patients with AECOPD in the emergency department and are consistent with those of previous studies [2, 19]. As confirmed in our study as well, most previous studies showed that the APACHE II and SAPS II scoring systems have similar capabilities for assessing disease severity and prognosis in ICU patients [20–24]. However, some studies found that SAPS II is superior to APACHE II [25], while others reported the opposite [26]. We believe that these reported differences in the systems are unlikely to reflect actual differences in prognostic ability; rather, they could stem from differences in candidate groups tested. A recent systematic review noted that, for ICU patients at admission, the SOFA score was equally effective for mortality prediction as the SAPS II score but was slightly inferior to the APACHE II/III score. Contrary to our findings, it also found that the sequential SOFA score is as effective as other model scores and that a combination of various models can significantly improve mortality prediction [19]. The survival of critically ill patients is closely related to the presence of multiple organ damage, and systematic monitoring of the SOFA score allows identification of patients with organ damage and provides real-time feedback on the effects of treatment. It is also the only system that can reflect the degree of organ damage. However, the clinical features of patients with AECOPD vary greatly and differ from those of critically ill patients with multiple organ failure. Therefore, the SOFA score is unable to reflect the severity of AECOPD in the early stage and is not suitable for assessment of early prognosis in patients with AECOPD.

Binary logistic regression analysis of the 28-day survival rate in the present study showed that the APACHE II and SAPS II scores significantly predict mortality (*P* < 0.05). The AUC of the APACHE II score was 0.899, while that of the SAPS II score was 0.862.

Additionally, the 28-day survival rate of critically ill patients with APACHE II scores ≥ 17 points was 56.76%, while that of patients with SAPS II scores ≥ 32 points was 52.78%. Thus, these two methods showed similarly good ability for predicting prognosis in critically ill patients with AECOPD in the emergency department.

Our study has some limitations: it included patients from a single center, and the sample size was small. Nonetheless, on the basis of our findings, we concluded that both APACHE II and SAPS II systems are useful for triage of patients with AECOPD who seek emergency care. Patients with AECOPD who have APACHE II scores ≥ 17 or SAPS II scores ≥ 32 points have a high mortality risk and should receive urgent attention. If possible, they should receive care in the respiratory ICU. Additionally, we believe that their survival rate may improve if their antibiotic therapy is adjusted to account for bacterial resistance, their fluid balance and nutrition are constantly monitored, any complications and comorbidities are identified and treated, and indications for mechanical ventilation are carefully observed.

## Figures and Tables

**Table 1:** Comparison of clinical data between the survival and death groups of patients with AECOPD.

	Survival group (*N* = 336)	Death group (*N* = 46)	Statistical magnitude (*t*/*χ*^2^)	*P* value
Gender (male/female)	214/122	26/20	0.19	0.505
Age (years)	62.80 ± 14.16	77.43 ± 10.96	−2.52	0.013
History of COPD (years)	13.8 ± 5.4	14.3 ± 5.6	0.95	0.35
Smoking (%)	189 (56.2)	27 (59.2)	0.38	0.54
BMI (kg/m^2^)	23.42 ± 2.81	22.71 ± 3.91	1.07	0.29
Temperature (°C)	36.59 ± 0.69	36.73 ± 0.84	−0.82	0.41
Pulse (times/min)	106 ± 15	112 ± 20	−1.27	0.22
Breathing (times/min)	21.2 ± 4.9	20.2 ± 3.1	0.96	0.33
SAP (mmHg)	139.4 ± 27.0	135.0 ± 28.8	0.72	0.47
DAP (mmHg)	76.9 ± 14.4	71.7 ± 19.7	1.54	0.12
MAP (mmHg)	97.8 ± 16.5	92.8 ± 20.2	1.30	0.19
GCS score	14.8 ± 0.8	12.0 ± 3.5	3.88	0.001
Renal inadequacy	74 (22.0)	16 (34.8)	3.657	0.056
Hypertension	96 (28.6)	20 (43.5)	4.252	0.039
Coronary heart disease	78 (23.2)	18 (39.1)	5.448	0.020
Diabetes	116 (34.5)	16 (34.8)	0.001	0.972
Cerebrovascular disease	68 (20.2)	14 (30.4)	2.495	0.114
Heart failure	54 (16.1)	30 (65.2)	56.969	<0.001
Arrhythmia	0 (0)	12 (26.1)	90.495	<0.001
Anemia	14 (4.2)	8 (17.4)	13.038	0.002
Gastrointestinal bleeding	8 (2.4)	2 (4.3)	0.614	0.344
Malnutrition	158 (47.0)	18 (39.1)	1.015	0.314
Fracture leading to bedridden status	4 (1.2)	4 (8.7)	11.115	0.001
Liver dysfunction	12 (3.6)	4 (8.7)	2.647	0.104
Tumor	2 (0.6)	12 (26.1)	74.470	<0.001
Pulmonary embolism	6 (1.8)	0 (0)	0.835	0.361
Thrombocytopenia	2 (0.6)	0 (0)	0.275	0.600

BMI, body mass index; COPD, chronic obstructive pulmonary disease; GCS, Glasgow coma scale; SAP, systolic arterial pressure; DAP, diastolic arterial pressure; MAP, mean arterial pressure.

**Table 2:** Comparison of severity-of-disease indicators between the survival and death groups of patients with AECOPD.

	*N*	APACHE II score	SAPS II score	SOFA score	MEDS score
Survival group	336	12.8 ± 3.2	24.0 ± 6.7	6.9 ± 4.4	1.77 ± 1.64
Death group	46	19.7 ± 5.2	37.0 ± 10.4	8.9 ± 5.8	3.52 ± 2.97
*P* value^#^		<0.001	<0.001	0.055	0.011
*P* value^*∗*^		<0.001	<0.001	0.028	<0.001

^#^ *P* value for *t*-tests; ^*∗*^*P* value for analysis of covariance model including age, gender, renal inadequacy, hypertension, coronary heart disease, heart failure, arrhythmia, anemia, fracture leading to bedridden status, tumor, and GCS score.

**Table 3:** Logistic regression analysis of the four severity-of-disease scoring methods.

	Variable	*P* value	Adjusted OR	95% CI of adjusted OR		AUC
				Lower	Upper	
Model I	APACHE II	<0.001	1.526	1.298	1.795	0.899
	Age	0.046	1.071	1.001	1.146	
	Coronary heart disease	0.001	11.420	2.779	46.938	
	Heart failure	<0.001	16.666	4.293	64.708	

Model II	SAPS II	<0.001	1.315	1.195	1.446	0.862
	Coronary heart disease	<0.001	20.018	4.913	98.664	
	Heart failure	<0.001	13.408	3.566	50.417	

Model III	AGE	0.031	1.067	1.006	1.132	0.661
	Hypertension	0.008	12.810	1.954	83.999	
	Heart failure	<0.001	19.187	5.302	69.432	
	Fracture keeping in bed	0.040	11.796	1.126	123.588	
	GCS scores	<0.001	0.656	0.498	0.865	

Model IV	GCS scores	<0.001	0.562	0.434	0.737	0.703
	AGE	0.031	1.067	1.006	1.132	
	Heart failure	<0.001	57.591	9.286	359.653	
	Tumor	0.017	17.304	2.548	>999	

Model V	APACHE II	<0.001	1.316	1.195	1.447	0.884
	SAPS II	<0.001	1.305	1.186	1.435	
	Coronary heart disease	<0.001	22.018	4.913	98.664	
	Heart failure	<0.001	13.408	3.566	50.417	

Model I included age, gender, renal inadequacy, hypertension, coronary heart disease, heart failure, arrhythmia, anemia, fracture leading to bedridden status, tumor, and GCS and APACHE II scores; Model II included age, gender, renal inadequacy, hypertension, coronary heart disease, heart failure, arrhythmia, anemia, fracture leading to bedridden status, tumor, and GCS and SAPS II scores; Model III included age, gender, renal inadequacy, hypertension, coronary heart disease, heart failure, arrhythmia, anemia, fracture leading to bedridden status, tumor, and GCS and SOFA scores; Model IV included age, gender, renal inadequacy, hypertension, coronary heart disease, heart failure, arrhythmia, anemia, fracture leading to bedridden status, tumor, and GCS and MEDS scores; Model V included age, gender, renal inadequacy, hypertension, coronary heart disease, heart failure, arrhythmia, anemia, fracture leading to bedridden status, tumor, GCS and APACHE II scores, and SAPS II; AUC, area under ROC curve; OR, odds ratios; CI, confidence interval.

**Table 4:** Comparison of the ROC areas of the APACHE II and SAPS II scores and their ability to predict the 28-day survival rate in patients with AECOPD requiring emergency critical care.

	AUC	SE	95% CI		*Z* statistics	*P* value
			Lower	Upper		
Model I	0.899	0.030	0.856	0.942	−0.706	1.519
Model II	0.862	0.043	0.811	0.912		

Model I included APACHE II scores, age, coronary heart disease, and heart failure; Model II included SAPS II, coronary heart disease, and heart failure; CI, confidence interval; SAPS, Simplified Acute Physiology Score; APACHE, Acute Physiology and Chronic Health Evaluation II; SE, standard error, AUC, area under the receiver operating characteristic curve.
